# Investigation of Iron Oxide Morphology in a Cyclic Redox Water Splitting Process for Hydrogen Generation

**DOI:** 10.3390/ma5102003

**Published:** 2012-10-23

**Authors:** Michael M. Bobek, Richard C. Stehle, David W. Hahn

**Affiliations:** Department of Mechanical and Aerospace Engineering, University of Florida, P.O. Box 116300, Gainesville, FL 32611-6300, USA; E-Mails: bobek86@ufl.edu (M.M.B.); rstehle@ufl.edu (R.C.S.)

**Keywords:** solar fuels, SEM, EDS, steam-iron process, water splitting, magnetite

## Abstract

A solar fuels generation research program is focused on hydrogen production by means of reactive metal water splitting in a cyclic iron-based redox process. Iron-based oxides are explored as an intermediary reactive material to dissociate water molecules at significantly reduced thermal energies. With a goal of studying the resulting oxide chemistry and morphology, chemical assistance via CO is used to complete the redox cycle. In order to exploit the unique characteristics of highly reactive materials at the solar reactor scale, a monolithic laboratory scale reactor has been designed to explore the redox cycle at temperatures ranging from 675 to 875 K. Using high resolution scanning electron microscope (SEM) and electron dispersive X-ray spectroscopy (EDS), the oxide morphology and the oxide state are quantified, including spatial distributions. These images show the change of the oxide layers directly after oxidation and after reduction. The findings show a significant non-stoichiometric O/Fe gradient in the atomic ratio following oxidation, which is consistent with a previous kinetics model, and a relatively constant, non-stoichiometric O/Fe atomic ratio following reduction.

## 1. Introduction

With the vast potential energy provided daily by the sun, roughly 5.6 × 10^24^ J/year, compared to the average energy of the world usage of 5 × 10^20^ J/year, many different avenues are being pursued in order to harness this untapped resource. Direct conversion of sunlight into electricity via PV cells provides an excellent addition of energy to our power grid, although this approach is variable and is limited by the band gap of the particular cell. Using concentrated sunlight for thermal processes is good on multiple scales from HVAC to power plant applications [1], but all solar power harnessing methods suffer from the potential disconnect of the sun’s availability and our societies energy usage. This leads to the importance of storing the solar-derived energy; for PV the direct conversion into electricity requires advanced battery technology, while for thermal processes some other means of storage must be used. One possible method for storage is using a portion of the collected solar energy to run thermo-chemical reactions with the purpose of creating fuel sources [2], such as splitting water to form hydrogen [3,4,5,6,7,8] or splitting of CO_2_ to form CO [9] or other hydrocarbon fuels [9,10], in what may collectively referred to as “solar fuels” [1,3]. Such an approach seeks to store the solar energy in the chemical bonds of the fuels, allowing for a stable, distributable, energy source that is compatible with existing infrastructures. 

With the motivation for the research of solar fuels stated above, the focus of this paper is in the examination of the oxide structure of the iron oxide layer developed during the redox process of cyclical hydrogen production via water splitting. The choice of iron as the metal to use is based off its natural abundance and high theoretical redox capacity per mass [11,12], although other metals and metal alloys have been suggested such as zinc oxides [9] and ferrite based alloys [13]. The two-step iron oxide-based redox reaction for water splitting has been studied in depth [9,14,15,16,17], while some difficulties of the associated reduction steps for iron are potentially mitigated by mixed metal ferrites [15,17,18,19], lowered reduction pressures, or chemically assisted reduction [20]. The current work focuses on the physical and chemical morphology of the resulting oxide layers in an iron-based redox cycle, a key item when designing process reactors for repeated redox cycling in combination with high efficiency production (*i.e.*, significant surface-to-volume ratios). Since the specific focus of this paper is the examination of the oxide layer, chemically assisted reduction, namely reduction via CO, was chosen to allow for precise and repeatable control of the level of reduction. 

## 2. Experimental Methods

### 2.1. Thermal Reactor Design

**Figure 1:**
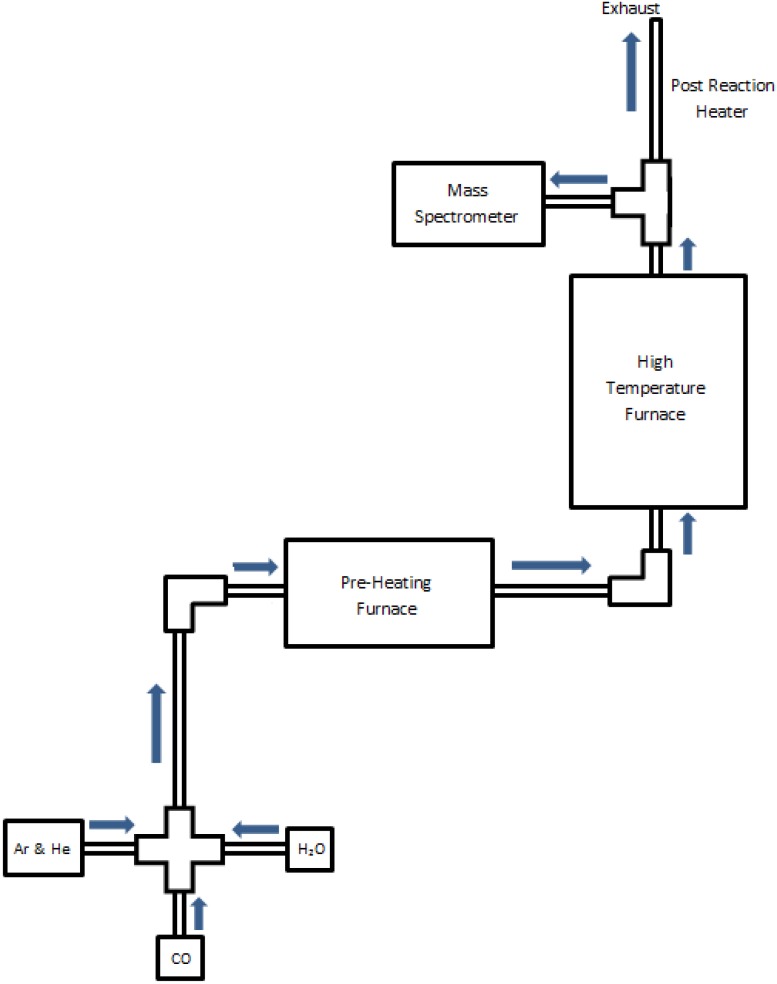
Monolithic reactor schematic designed for redox process.

A laboratory-scaled reactor was used for precise control and monitoring of the iron-steam oxidation reaction and reduction processes during single cycle and multi-cycle tests. The flow of all gases was controlled by mass flow controllers (Alicat Scientific), and the reactor temperature was controlled and monitored by SentroTech (stt-1700C-1.5-18; stt-1200C-2-12) tube furnaces. All experiments were conducted using a constant surface area monolith, as created using 6.35 mm O.D. elemental iron rods (Surepure, 99.5% Fe) at a fixed length of 30.5 cm. The stt-1200 furnace was used to preheat the carrier gases as well as for steam generation, with the liquid water supplied precisely via a syringe pump (BASi). The water was supplied by a 1.59 mm I.D. stainless steel tube coiled within a 12.7 mm I.D. stainless steel situated within the furnace test section. The annular tube had inert gas flows of argon and helium, into which the steam is injected at the exit of the steam tube coil. The gases (steam and inert gases) are preheated to a temperature of 750 °C, and then flow through a heated transfer section into the main reactor furnace. The inert gases allow for the variation of concentration, and for quantitative monitoring of the reactor output by a quadrupole mass spectrometer (MS) (Hiden model HPR-20). To create the annular reactor space test-section, the iron test rod was positioned axially within a 12.7 mm I.D. tube (705 Inconel) that was situated within the stt-1700 furnace test section. Control experiments with no iron monolith showed no hydrogen production from the Inconel housing. Thermocouple probes were used to both calibrate and validate the reactor test bed temperature against the furnace controller settings. The reactor temperature was then assumed representative of the substrate reactive layers under reacting conditions, given the significant thermal inertia of the iron monolith. During the oxidation phase experiments, the iron and the steam in the gas flow react to form an iron-oxide layer and release hydrogen, as described in detail in a recent publication [21]. During the chemically-assisted reduction experiments, the steam flow was terminated, and CO was introduced into the gas flow to react with the iron-oxide layer. During redox cycling experiments, the flow of the gases and the steam was set and controlled through a LabView VI that was connected to the flow controllers and syringe pump such that the timing was consistent on a cycle-to-cycle basis. A schematic of the reactor can be seen in Figure 1.

### 2.2. Data Acquisition

**Figure 2:**
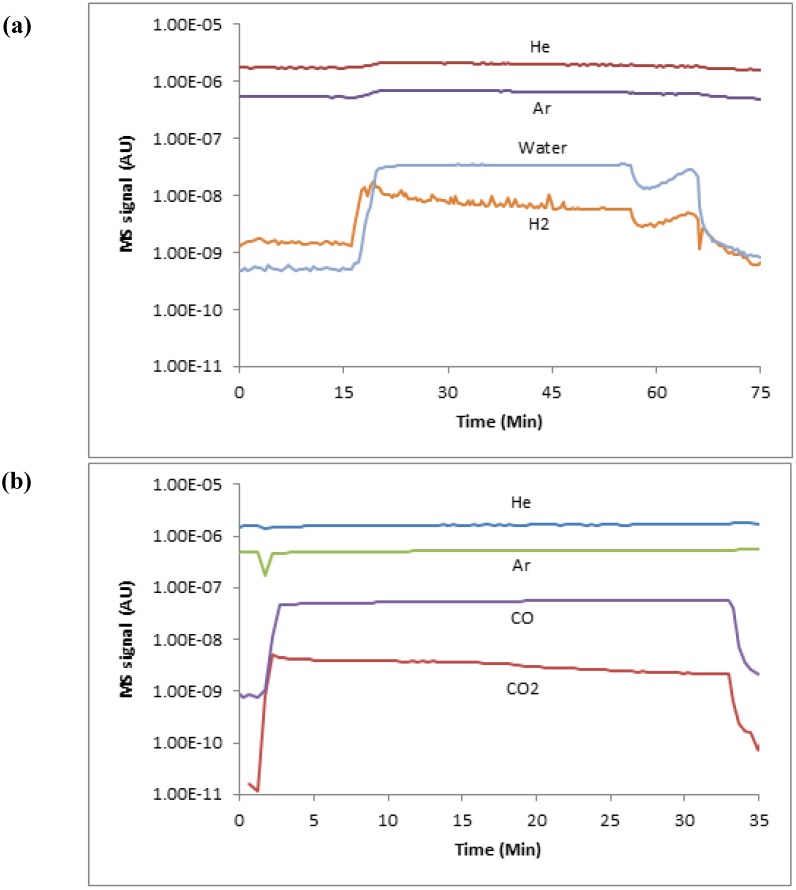
MS traces of initial oxidation cycle data for a reactor temperature of 873 K (**a**), and of initial reduction cycle data for a reactor temperature of 873 K (**b**).

During each experiment, the output gas species concentrations were quantified using the MS, with all gas sampling accomplished via a heated quartz capillary tube. The MS was directly calibrated, and all experimental data were reduced using the flow of argon as an internal standard. The calibration was done using a series of known gas flow rates and mixtures without a reactive sample present; the signal ratio of each gas to argon was then used to build a calibration curve. In addition, the known Ar/He ratio was used to validate each experimental run for additional quality control. Finally, all hydrogen signals were corrected for water fragmentation based on a direct calibration using the current MS detector settings and relevant steam flow. Figure 2 shows typical MS readings for an initial oxidation and reduction cycle.

After each experiment was performed, and the entire reactor, including the iron redox sample, was allowed to cool under inert gas flow, after which the rod was removed and stored under inert gas prior to analysis. For chemical and morphological examination, the test rod was sectioned using a diamond saw, and set in epoxy (EpoxiCure 20-8130-032) to prep for polishing, and eventually SEM and EDS (energy dispersive X-ray spectroscopy) imaging. The SEM and EDS images were obtained with a JSM 6400 and a S3000N SEM, with a number of SEM images and EDS scans recorded for each sample. The SEM images provide details of the iron bulk and oxide interface behavior, and EDS allows for composition information to be determined. Specifically an EDS line scan across the oxide layer was used to characterize and quantify the oxide behavior for multiple runs under different conditions and end states. The EDS O/Fe atomic data were quantified with pure standards (pressed pellets) of Fe_2_O_3_ and Fe_3_O_4_ using a two-point linear calibration curve. 

### 2.3. Experimental Procedure

The redox experiments were performed over a wide range of parameters, with each test initiated with an initial oxidation process to build a base oxide layer (primarily Fe_3_O_4_ as reported previously [21]) with gas flow rates of argon at 200 mL/min and helium at 100 mL/min, and the liquid water supplied at 12.5 µL/min. The temperature range for this initial oxidation was 675 to 875 K, with a run time of 30 to 60 min. This range was chosen for the stability of the oxide layer developed [21]. The reduction step was done in the temperature range of 675 to 875 K, with the flow rate of CO at 12.5, 25, 50, and 100 mL/min. The number of redox cycles was also varied from a single cycle, to a range of 25 to 50 cycles. Roughly half of the samples were terminated after a reduction step, and half of the sample experiments were terminated following an oxidation step. Prior to each experiment, a sample iron rod was sanded using a successive series of grit papers (320, 600, and 1200), then carefully cleaned with acetone to produce a polished rod free of any surface oxidation. Following surface preparation, the rods were inserted into the ambient temperature reactor that was pre-purged with inert gasses. The insert gas flow was maintained while the reactor was heated to the desired operating temperature. While the furnace was raised to the set temperature, the MS sampling was initiated to record the output flow and verify only inert gasses were flowing (*i.e.*, no oxygen or water present). Once the desired set temperature was reached, the initial oxidation for the base oxide layer was initiated as described above, and then the desired redox cycling would begin and continue to the desired number of cycles and terminating condition (*i.e.*, oxidation or reduction). After a set number of cycles, the experiment would be allowed to cool down under an inert gas flow as described above. 

## 3. Results

### 3.1. Termination Following Oxidation

**Figure 3:**
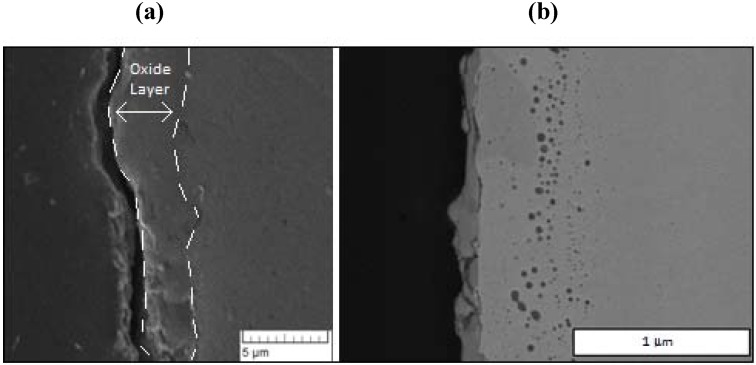
Scanning electron microscope (SEM) images of oxide samples terminated after oxidation: (**a**) After 24 cycles at a reactor temperature of 685 K; and (**b**) after 21 cycles at 765 K.

Representative SEM images of the oxide layer films for two cycles terminated on oxidation are shown in Figure 3 for reactor temperatures of 685 and 765 K. The lower temperature sample corresponds to 24 redox cycles, while the higher temperature sample corresponds to 21 redox cycles. 

From these typical images, the general structure of the oxide layer and its approximate thickness are readily observed. After repeated experimentation, the oxide thickness revealed considerable variability from run to run, as well as around the perimeter of a given specimen. For all experiments, the average over all samples (N = 21) was found to be 5 microns with a standard deviation of 3 microns. As discussed in our previous study [21], the large differences are attributed primarily to either spallation of the oxide layer or to fracture during polishing. While the EDS data profiling is discussed below, these films are considered primarily as magnetite, Fe_3_O_4_, based on previous EDS and Raman spectroscopy [21].

**Figure 4:**
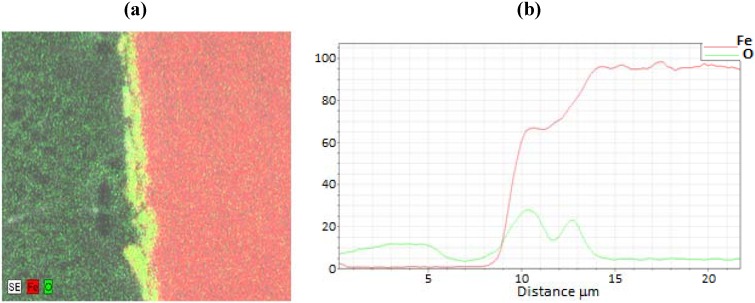
(**a**) Electron dispersive X-ray spectroscopy (EDS) full scan image and (**b**) line scan across the oxide film for the 685 K sample of Figure 3.

A representative EDS image is shown in Figure 4 for a sample film terminated following oxidation for the experiment conducted at 685 K. EDS line scans were recorded across each analyzed oxide film specimen and the O/Fe atomic ratios were calculated. The corresponding EDS line scan for the EDS image map of Figure 4 is also included in the figure. As observed, the iron signal is essentially zero at the outer surface (z = 5 µm), and becomes constant at the inner bulk iron surface (z = 14 µm), giving a total oxide thickness of about 5.5 µm for this film. In order to quantify and compare the oxide behavior formed from the different cycle parameters, the oxide layer thickness was normalized for each sample from 0 to 1 over the full thickness, with zero corresponding to the outer surface, and one corresponding to the start of the bulk iron substrate (*i.e.*, oxide layer inner surface). This allowed for comparisons to be made more intuitively between different experimental runs. 

**Figure 5:**
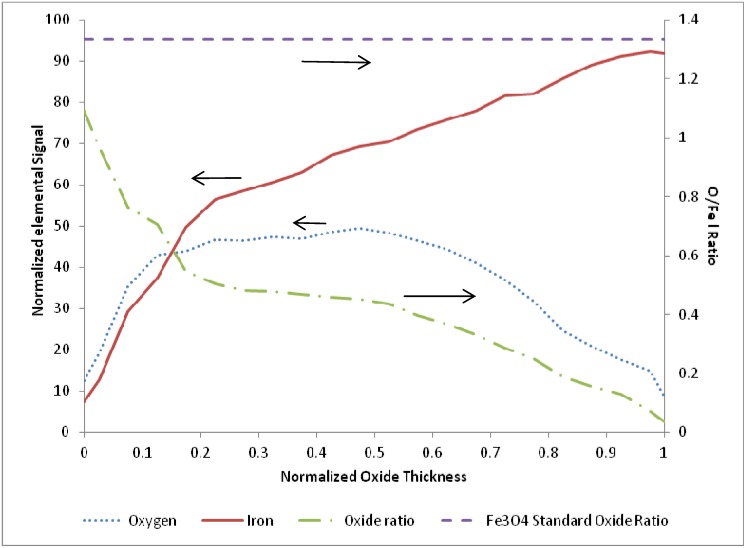
Average of 11 EDS line scans through the oxide layer from samples terminated after an oxidation step, with 0 corresponding to the outer edge of the oxide layer and 1 corresponding to the iron-iron oxide interface.

After examination of the oxidation terminated SEM and EDS data over the entire temperature range of 675 to 875 K, there was no clear trend observed with temperature or cycle number. Therefore, the oxidation sample normalized line scans were pooled and averaged (N = 11) to produce a representative O/Fe profile. The resulting average species profiles are presented in Figure 5. Several interesting findings are noted in the average oxidation terminated profile. First, the O/Fe atomic ratio is very near the stoichiometric ratio for Fe_3_O_4_ at the outer surface, and decays monotonically to a near-zero atomic ratio at the inner oxide/bulk iron interface. Such a quantitative profile of the oxide ratio is consistent with our previous findings of magnetite at the surface via Raman spectroscopy, and of an O/Fe gradient consistent with a diffusion model based on a Cabrera-Mott kinetics mechanism [21]. As the oxide layer grows, there is diffusion of oxygen and iron species, resulting in a continuous gradient and non-stoichiometric iron oxide throughout the length of the oxide layer. This finding stands in contrast to conceptual models based on uniform, idealized stoichiometric (e.g., Fe_3_O_4_) oxide layer growth.

### 3.2. Termination Following Reduction

**Figure 6:**
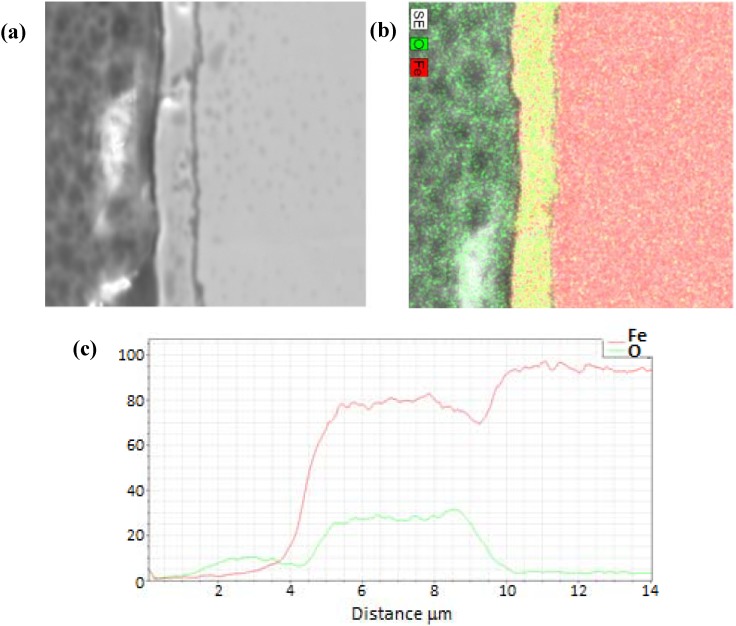
Sample terminated after reduction at a temperature of 765 K, including (**a**) SEM image, (**b**) EDS full scan image, (**c**) and line scan across oxide.

A representative SEM and corresponding EDS image of an oxide layer film terminated following CO-assisted reduction are shown in Figure 6 for a reactor temperature of 765 K. The sample corresponds to termination after 22 redox cycles. As observed in the corresponding EDS image, the iron signal drops markedly to zero at the outer surface (z = 5 µm), and becomes constant at the inner bulk iron surface (z = 0 µm), giving a total oxide thickness of about 5 µm for this film. In order to quantify and compare the oxide behavior formed from the different cycle parameters, the oxide layer thickness was normalized for each sample from 0 to 1 over the full thickness, with zero corresponding to the outer surface, and one corresponding to the start of the bulk iron substrate, in an identical manner described above as with the oxidation terminating experiments.

**Figure 7:**
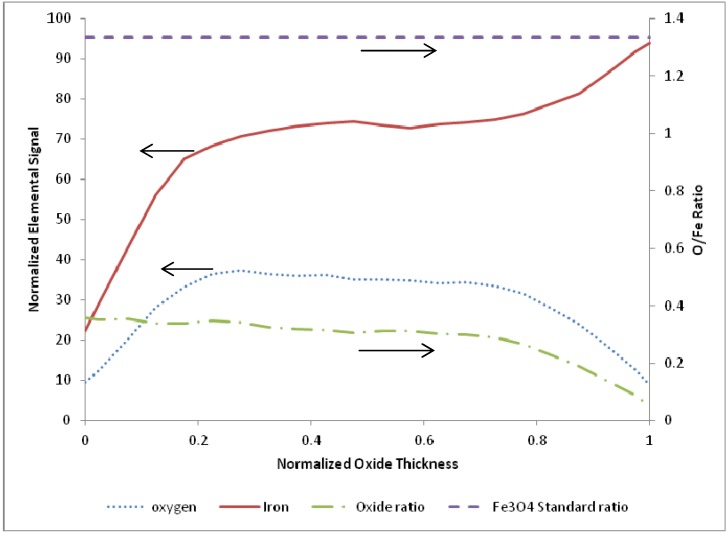
Average of 10 EDS line scans through the oxide layer from samples terminated after ending on a reduction step, with 0 corresponding to the outer edge of the oxide layer and 1 corresponding to the iron-iron oxide interface.

As observed with oxidation-terminating films, examination of the reduction-terminating data over the temperature range of 675 to 875 K revealed no clear trend. Table 1 presents the individual experimental conditions and corresponding oxide layer thickness for the reduction runs. As with the oxidation data, the reduction sample normalized line scans were pooled and averaged (N = 10) to produce a representative O/Fe profile, which is presented in Figure 7. 

As observed in the figure, in the outermost 75% of the oxide layer the O/Fe atomic ratio is very near a constant value equal to about 25% of the expected stoichiometric ratio for Fe_3_O_4_. This near-constant value does diminish over the innermost 25% of the oxide thickness layer, where it is characterized by reduced oxygen content to values similar to the oxidation-terminated layers. This latter trend is further elaborated on below. The behavior of the O/Fe atomic ratio for CO-assisted oxidation under this range of temperature data suggests that a minimum oxygen content is maintained within the film relative to the diffusive mobility of the oxygen and/or CO.

**Table 1:** Oxidation terminated experimental conditions, number of redox cycles, and corresponding measured average oxide layer thickness.

Temperature (K)	Number of Cycles	Oxide Thickness (µm)
873	1	5.9
873	1	3.5
796	21	4.8
796	21	9.8
796	21	3.1
673	22	8.8
673	22	5.6
761	26	4.9
761	26	5.9
761	26	12.4

## 4. Discussion and Conclusions

**Figure 8:**
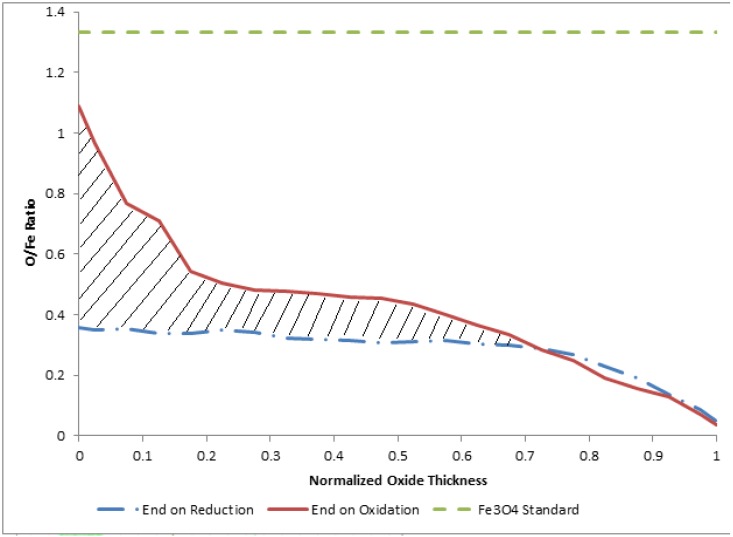
Comparison of relative oxygen content through the oxide layer for films terminated following reduction and films terminated following oxidation. The horizontal line represents the standard for Fe_3_O_4_. In the figure, 0 corresponds to the outer edge of the oxide layer and 1 corresponds to the iron-iron oxide interface.

As discussed in the motivating comments above, a successful water-splitting process using a reactive metal redox cycle must operate efficiently over many (e.g., thousands) of redox cycles. We note in this study and in our previous work that iron oxide, namely Fe_3_O_4_, spalls due to structural incompatibilities between the bulk and oxide states, and appears to be most stable at a layer thickness on the order of 5 to 10 µm. Given the equilibrium thermodynamics of the various iron oxide states [9,15,22,23], combined with the physical morphology and stability of the oxide layers, the ability for total (*i.e.*, complete) stoichiometric reduction will be difficult to achieve in any practical reactor with high surface-to-volume ratios (e.g., fine grain powders) and rapid cycling times. Considerable attention has been given to reactor design for iron-based redox systems, although the role of resulting oxide film structures, as related to oxidation and reduction kinetics has been somewhat overlooked [24]. To gain additional insight into this important facet of hydrogen production via iron redox systems, we present in Figure 8 the average O/Fe atomic profiles obtained for our oxidation-terminated and reduction-terminated oxide films. 

The hatched area, which extends from the outer surface to about 70% of the full oxide layer thickness, is considered as representative of the available oxygen for redox participation. In other words, this area is representative of the oxygen atoms added to the layer during hydrogen production (*i.e.*, oxidation) and removed from the oxide layer during reduction. Here we note the non-stoichiometric nature of the available oxidation, which we believe results from the diffusive-controlled kinetics and therefore non-equilibrium oxide films produced during redox cycling. 

As a first-order approximation, we have quantified the available oxygen, and therefore hydrogen production, corresponding to the shaded area of Figure 8 for our current average oxide film thickness of 4.6 µm. In other words, integrating the difference between to two average atomic ratios yields the available oxygen and iron for water splitting over one complete redox cycle, and therefore an estimate of the available hydrogen production. This calculation yields about 1E-5 g/cm^2^ of hydrogen per redox cycle using only this available oxygen. This value is in good agreement (factor of 2) with the current integrated MS data for a single redox cycle (e.g., see Figure 2). Such a value speaks to the importance of reactor kinetics, as well as the resulting oxide film morphology and chemical composition for implementation of water splitting concepts in solar-thermal reactors using an iron oxide intermediary. It is envisioned that further quantitative study of the oxidation and reduction processes with regard to native oxide layer structure and composition will help advance the design and optimization of efficient solar fuel production via reactive metal redox cycles. 
